# Experimental and Numerical Analysis of the Deformation Behavior of Adaptive Fiber-Rubber Composites with Integrated Shape Memory Alloys

**DOI:** 10.3390/ma15020582

**Published:** 2022-01-13

**Authors:** Felix Lohse, Karl Kopelmann, Henriette Grellmann, Moniruddoza Ashir, Thomas Gereke, Eric Häntzsche, Cornelia Sennewald, Chokri Cherif

**Affiliations:** Institute of Textile Machinery and High Performance Material Technology, Technische Universität Dresden, 01062 Dresden, Germany; karl.kopelmann@tu-dresden.de (K.K.); henriette.grellmann@tu-dresden.de (H.G.); moniruddoza.ashir@tu-dresden.de (M.A.); thomas.gereke@tu-dresden.de (T.G.); eric.haentzsche@tu-dresden.de (E.H.); cornelia.sennewald@tu-dresden.de (C.S.); Chokri.Cherif@tu-dresden.de (C.C.)

**Keywords:** shape memory alloy, fiber-rubber composite, simulation, smart material

## Abstract

Fiber-reinforced rubber composites with integrated shape memory alloy (SMA) actuator wires present a promising approach for the creation of soft and highly elastic structures with adaptive functionalities for usage in aerospace, robotic, or biomedical applications. In this work, the flat-knitting technology is used to develop glass-fiber-reinforced fabrics with tailored properties designed for active bending deformations. During the knitting process, the SMA wires are integrated into the textile and positioned with respect to their actuation task. Then, the fabrics are infiltrated with liquid silicone, thus creating actively deformable composites. For dimensioning such structures, a comprehensive understanding of the interactions of all components is required. Therefore, a simulation model is developed that captures the properties of the rubber matrix, fiber reinforcement, and the SMA actuators and that is capable of simulating the active bending deformations of the specimens. After model calibration with experimental four-point-bending data, the SMA-driven bending deformation is simulated. The model is validated with activation experiments of the actively deformable specimens. The simulation results show good agreement with the experimental tests, thus enabling further investigations into the deformation mechanisms of actively deformable fiber-reinforced rubbers.

## 1. Introduction

Adaptive structures have been an increasingly popular field of research in recent years, with the numbers of publications and patents rising significantly since the year 2000 [1]. An adaptive structure is capable of reacting to external influences with changes in its properties [2]. Due to their wide range of properties, mechanisms, and thus possible applications, they offer a high potential for innovations in many fields of technology, such as aerospace [3], biomedicine [4], and robotics [5]. Adaptive structures are usually driven, controlled, or supported by smart materials that act as structurally integrated actuators and sensors. Smart or intelligent materials are materials capable of changing their specific properties under the influence of external electrical, magnetic, or thermal fields. Actuators based on smart materials have, therefore, been the subject of ample research in recent years [6]. Typical examples of smart materials are piezoceramics [1], electrostrictives [7], and shape memory alloys [8].

Recently, shape memory alloys (SMAs) have emerged as promising candidates for creating adaptive structures. Their ability to react to thermal stimuli with reversible shape changes allows users to reduce the complexity, size, and cost of mechanical systems by replacing conventional actuators, such as electrical motors, hydraulics, or pneumatics [9]. They offer high activation forces (20–60 N for contractile wire actuators) and deformations (4–6% contraction) in comparison to other popular smart actuators (large in comparison to Piezo but smaller than shape memory polymers). SMAs are, therefore, predestined as a basis for the development of functionally integrated adaptive structures with high functional density. Fiber-reinforced polymers (FRPs) are well suited for functional integration due to their freedom of design provided by the combination of matrix and reinforcement fibers, which can be tailored to specific applications [10]. The most common FRPs are based on thermoset resins, which possess high stiffness and thus allow comparatively low structural strain [11,12]. Therefore, composite structures with a highly stretchable rubber matrix are a promising basis for adaptive structures capable of large deformations due to their high reversible strains [13,14,15,16]. Advances in textile technology [17,18] enable the tailoring of reinforcement textiles to the specific requirements for such highly deformable fiber-rubber composites (FRCs) and the integration of actuators such as wire-shaped SMAs directly into the fabric. Thus, structures capable of active deformations, further denoted as interactive fiber-rubber composites (IFRCs), are created. Due to their highly flexible deformation capabilities and adjustable mechanical properties, IFRCs represent a highly promising new group of smart composites for future innovative applications in the fields of soft robotics and human–machine interactions [19,20,21]. 

For the development of practical applications, a comprehensive understanding of the new IFRC material behavior and the interactions of the separate components is required. To date, no feasible approaches for dimensioning and investigating the deformation behavior of IFRC exist. Therefore, a simulation model capable of recreating and predicting the active deformation behavior of real specimens is required. In this work, multilayer flat knitting is used to develop biaxially reinforced multilayered knitted fabrics with integrated SMA wires. The fabrics are manufactured into FRC and IFRC specimens with vacuum-assisted resin infusion (VARI) technology and tested regarding their deformation behavior. A novel mesoscopic model within the framework of the finite element method (FEM) for predicting the structural IFRC behavior is developed and validated with the results from the experiments, providing a basis for future developments of more complex IFRC structures and applications.

### 1.1. State of the Art

#### 1.1.1. Shape-Memory-Alloy-Driven Actuation Mechanisms

SMAs are capable of regaining their initial shape after an applied deformation through heating to a value above the material-specific transformation temperature. This property is based on their ability to perform a diffusionless solid-phase transformation between two distinctive microscopic phases—the martensite (M) phase, which is stable below the transformation temperature range, and the austenite (A) phase, which is stable above the transformation temperature range [1]. The switch between the two phases is characterized by the respective start and end temperatures of the forward and backward transformation:

1. The start and finish temperatures of austenite forming A_s_ and A_f_ (heating).
2. The start and finish temperatures of martensite forming M_s_ and M_f_ (cooling).

Several effects result from these thermomechanical properties—most prominently the one-way shape memory effect (OWSME), which will be the focus of this work. For more details on other SMA-specific effects (two-way shape memory effect and superelasticity) and the complex material behavior of SMAs, refer to, for example, [8,9,22].

**Figure 1:**
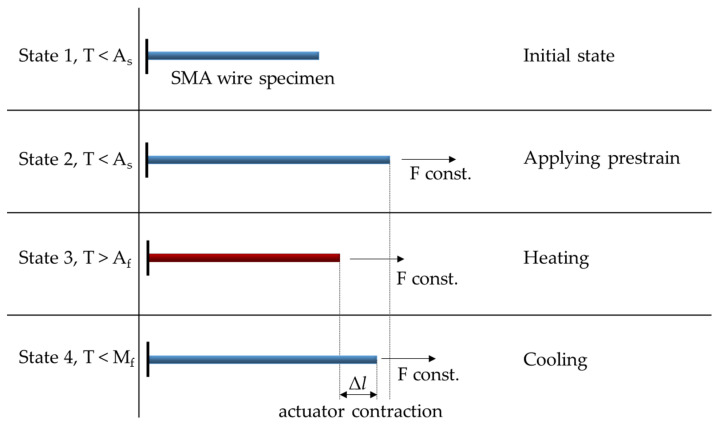
Basic principle of the one-way shape memory effect.

The basic principle of the OWSME is displayed in Figure 1. First, an SMA wire is transferred from the undeformed initial state 1 with twinned martensite to the deformed state 2 by a mechanical load. Thus, the martensitic structure is detwinned and is now oriented. By heating the wire to a value above A_f_, the microstructure transforms from martensite to austenite. Since austenite possesses a much higher stiffness and lacks the property of detwinning, a contraction of the wire now occurs toward the undeformed state 1, which, however, is not fully reached due to the external load. Instead, state 3, which is afflicted with residual deformation, is adopted, corresponding with the stress–strain behavior of the austenitic SMA. The extent of this contraction, i.e., the difference between states 2 and 3, is referred to as the actuator contraction Δ*l*. Upon cooling to a value below the transformation temperature M_f_, the microstructure of the wire transforms back into martensite. If no external force was applied here, the wire would remain in the geometric state 3 or, in the case of a slightly trained material, deform to a small extent toward state 4. However, due to the force still applied, which now acts as a resetting force, the wire is stretched again and assumes state 4. Thus, by applying temperature and using a setup that provides a resetting force, it is possible to repeatedly switch back and forth between states 3 and 4. The usable actuator contraction of a typical nickel–titanium (NiTi) SMA usually is in a range of 4 to 8% based on the initial length. For applications requiring long-term stability, thermal stabilizing methods are sometimes used that reduce the usable contraction to 4–5%. Due to their large actuation forces and multitude of intrinsic effects, SMAs are widely used in different fields of technology, such as aerospace [23,24], medicine [25,26], automotives [27], and soft robotics [14,28]. 

#### 1.1.2. Fibe-Rubber Composites (FRCs)

Thermoset fiber-reinforced polymers (FRPs) possess high stiffness and strength. The reinforcement fibers carrying tensile loads along their longitudinal axis and the resin matrix provide stability between the fibers and take loads perpendicular to the fibers, as in pressure or shear load cases [29]. In contrast, fiber-rubber composites (FRCs) combine a highly stretchable elastomer matrix with reinforcing fibers, giving them highly anisotropic properties—usually high strength and stiffness in the fiber direction and high, reversible deformability in the perpendicular direction. The soft rubber matrix is unable to support the reinforcement fibers in load cases perpendicular to the fiber direction. Therefore, FRCs are typically used in applications with one-directional loading, such as car tires, pressure hoses, and conveyor belts [29].

Using the capabilities of textile technology, the mechanical properties of the FRCs can be tailored in such a way that they match the properties of the SMA actuators and thus enable large and reversible deformations. The multilayer knitting technology [17,18,30] enables the user to tailor the properties of a reinforcement textile to their respective requirements and integrate additional functional components into the fabric, offering a high scope for design while requiring a small machine configuration and programming effort in comparison to other textile processes. 

#### 1.1.3. Deformation Mechanism of Interactive Fiber-Rubber Structures

**Figure 2:**
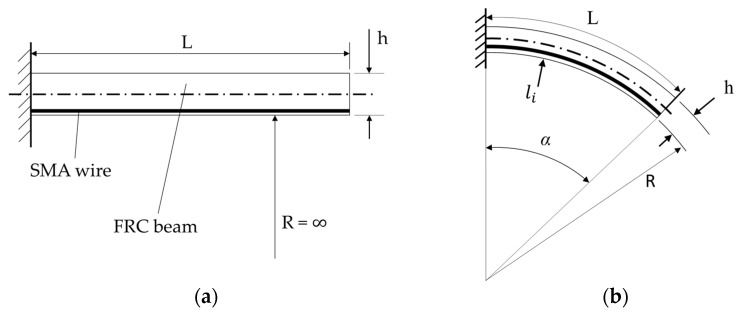
Geometrical description of the bending beam mechanism with a straight beam (**a**) and in bent shape (**b**).

The SMA-driven IFRC mechanism of this work is based on the bending beam, as depicted in Figure 2a. An SMA wire is inserted into a symmetrical beam structure and fixed at its ends. The wire is able to move freely along its length direction. The position of the SMA with respect to the thickness is eccentric from the neutral axis of the beam. After thermal activation, the SMA contracts and begins to deform the structure by acting with a force on the fixation points, pulling them toward each other and deforming the beam structure into a bent shape (Figure 2b).

The two-dimensional shape of a deformed beam with an integrated SMA is assumed to match a circular arc, as depicted in Figure 2b. The length of the beam *L* is assumed to stay constant throughout the deformation process. The length *l_i_* of the inner arc corresponds to the length of the SMA wire under a given contraction Δl with

(1) $$\Delta l=1-\frac{l_{i}}{l_{0}}$$

where the initial length of the SMA $l_{0}$ is assumed to be equal to the beam length *L*. For a given thickness *h* and length *L*, the resulting geometry is calculated with

(2) $$\alpha =\frac{360}{\pi \cdot h}\cdot \Delta l\cdot L$$

(3) $$R=\frac{h}{2\cdot \Delta l}$$

Here, α is the aperture angle and *R* denotes the radius of the arc. This geometrical consideration assumes the maximum possible deformation for a given SMA contraction neglecting mechanical interactions of the structural stiffness that reduce the effective SMA contraction. Two basic design principles can be derived:

1. Thinner structures enable larger deformations (i.e., lower values of *h*).
2. Longer structures enable larger deformations (i.e., higher values of *L*).

**Figure 3:**
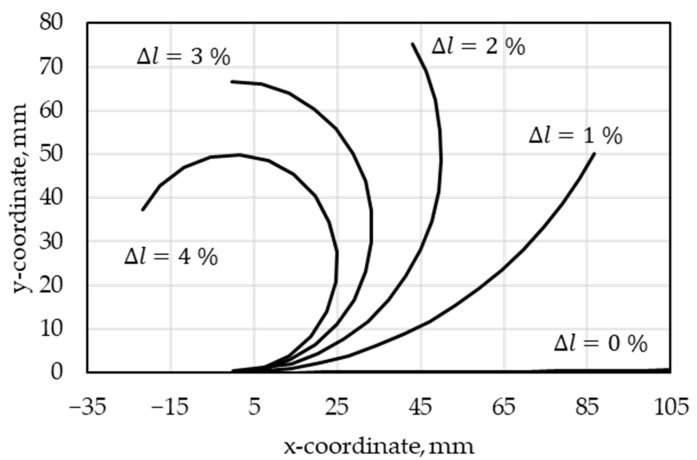
Geometrical prediction of achievable deformations for an SMA-actuated bending beam with dimensions 105 × 80 × 2 mm³ and different SMA contractions Δ*l*.

For an exemplary beam with dimensions 105 mm × 80 mm × 2 mm, the maximum deformation was calculated for SMA deformations of up to 4%. The resulting theoretical geometries are displayed in Figure 3.

Wang et al. [31] described the following phenomenon: Once the structure starts bending, the SMA wire comes into contact with the surrounding structure along its length and begins acting with a line force onto its containment channel. With progressive deformation, both the force onto the fixation points and the line force onto the channel incrementally change their directions correlative to the shape change of the structure. Furthermore, the material behavior of the SMA needs to be taken into account for mechanically accurate deformations, which introduces further complexity into the equations. Therefore—as shown, e.g., by Wang et al. [31], Icardi et al. [32], and Marschner et al. [33]—mathematical descriptions of these interactions are difficult to solve analytically. Using a fiber-rubber structure with anisotropic hyperelastic properties significantly increases the complexity of the mechanism, making an analytical solution impractical. 

Furthermore, as was investigated by Drissi-Habti et al. [34,35], an SMA actuator integrated into a composite can be understood as a “defect” to the structure and must, therefore, be integrated in such a way that the harm done to the composite is minimized, e.g., by miniaturization, optimizing physical operating conditions, and reducing the overall invasiveness of the actuator. Thus, finite element analysis is required to capture the relevant components with their properties as well as their interactions, estimating their influences on the structural deformation behavior and optimizing the composite topology toward an optimal actuator integration while minimizing the harm inflicted upon the overall structure.

## 2. Materials and Methods

### 2.1. Experimental Investigations

#### 2.1.1. Materials

The SMA material used in this work was NiTi wire with a diameter of 0.3 mm, provided by SAES Getters (20045 Lainate (Milan), Italy). The material was obtained in a pre-strained state, able to initiate the OWSME by thermal activation and thus providing the required condition for application as structurally integrated actuators. As the rubber matrix material, the two-component liquid silicone polydimethylsiloxane (Sylgard 184™) was used. As fiber reinforcement, glass fiber (GF) rovings with 410 tex and twisted GF twin-yarns with 2 × 136 tex were used.

#### 2.1.2. Development of Functionalized Reinforcing Fabrics

**Figure 4:**
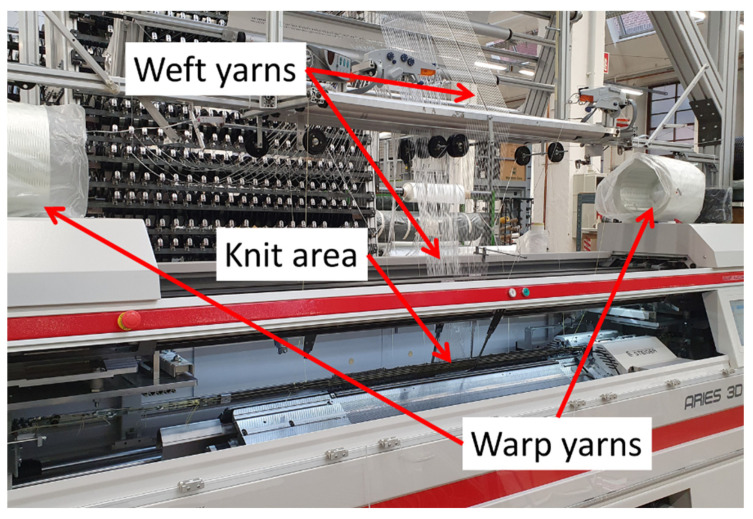
Modified Steiger Aries.3^®^ flat knitting machine.

**Figure 5:**
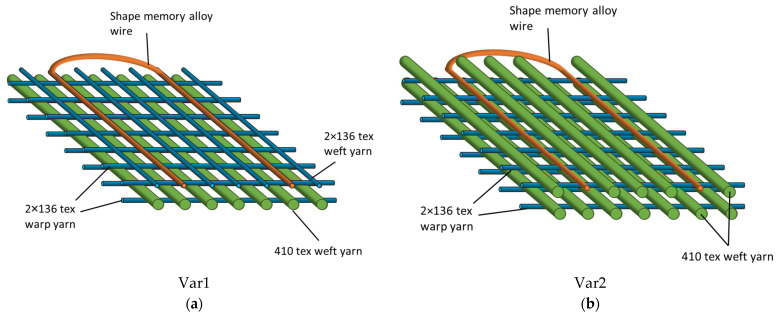
Concept of the biaxially reinforced multilayer knitted fabrics with (**a**) thin 2 × 136 tex glass fibers in the top layer and (**b**) thicker 410 tex glass fiber rovings in the top layer.

Due to their high thermal stability, favorable mechanical properties, advantageous price–performance ratio, and commercial availability, GFs are well suited for usage in IFRCs [36]. A customized Steiger Aries.3^®^ flat knitting machine (Figure 4) with a segmented take-up [17] was used to manufacture biaxially reinforced multilayered knitted fabrics with integrated SMA actuators. GF rovings with 410 tex were used as weft yarns, and twisted GF twin-yarns with 2 × 136 tex were used both as weft and warp yarns (Figure 5). The textile was constructed in such a way that a layer of reinforcement fibers was placed in the direction of the bending deformation, offering a defined stiffness against the SMA wires during activation. Polytetrafluoroethylene (PTFE) tubes were inserted into the fabric during the knitting process, into which the SMAs were inserted. That way, the number of SMAs within a sample could be varied even after the composite manufacturing. The tubes holding the SMA wires were placed outside of the neutral axis of the textile by placing them in a second layer, which additionally contained GF with reduced stiffness (Var1). A second variant (Var2) was produced, which contained the same 410 tex GF in both layers. At the end of one weft row, the SMA wire was redirected in a loop and inserted into a subsequent weft row, forming a meandering arrangement. The concepts of both fabric variants are displayed in Figure 5a,b, respectively.

#### 2.1.3. Composite Development and Manufacturing

**Figure 6:**
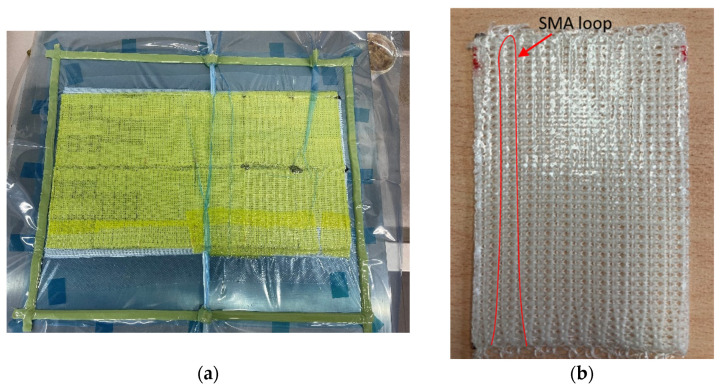
VARI process (**a**) and IFRC specimen (**b**).

A vacuum-assisted resin infusion (VARI) process was used for the manufacturing of the composite specimens. Cuttings of the GF knit (120 × 80 mm²) were placed onto a metal plate and covered with a multilayer buildup as listed in Table 1. Figure 6a shows the VARI layup.

The silicone was degassed before the infusion process. This step is required to avoid air bubble formation. After the infusion, the specimen was cured for 48 h at room temperature until it was unwrapped (Figure 6b). To achieve defined thicknesses, the cured specimens are placed in a pouring mold and additional silicone is poured on top. 

### 2.2. Material and Composite Characterization

To achieve a comprehensive understanding of the material properties as well as obtain relevant mechanical and thermomechanical properties of the IFRC components and the composite itself, characterization experiments of all components were conducted. Due to the SMA wires possessing the most complex material behavior, the characterization experiments were the most comprehensive. Here, the tensile properties below and above the transformation temperature range had to be obtained, as well as the temperature-dependent shape change behavior, including the start and end temperatures of the transformation process. For reinforcement fibers and silicone rubber, the tensile properties were the most relevant and were obtained from tensile tests. Since the primary deformation mode of the composite during SMA-driven activation is bending and the SMA wires are acting against the structural bending stiffness, the composite bending behavior was the most significant mechanical property for calibrating the simulation model. Therefore, four-point-bending tests were conducted on composite specimens without SMA wires, to be used for comparison with the simulation model in Section 3.4.

#### 2.2.1. SMA Wire Characterization

The basic properties of the material were obtained by the following experiments:

- Differential scanning calorimetry (DSC) [37].
- Isothermal tensile test at room temperature (below A_s_).
- Isothermal tensile test above 130 °C (above A_f_).
- Uniaxial pre-strain and free recovery (UPFR) test.

In DSC measurements, a crucible with sample material and an empty reference crucible are heated or cooled uniformly in a defined manner. From the comparison of the heat flows of both crucibles, statements are made about the transformation processes in the sample material. For the SMA material, the four transformation temperatures M_s_, M_f_, A_s_, and A_f_ are determined. The DSC measurement was conducted with a DSC Q2000 by TA Instruments (Dallas, TX, USA). The SMA material was heated twice from 0 to 180 °C. In this way, the pre-strain within the material was removed during the first cycle.

**Figure 7:**
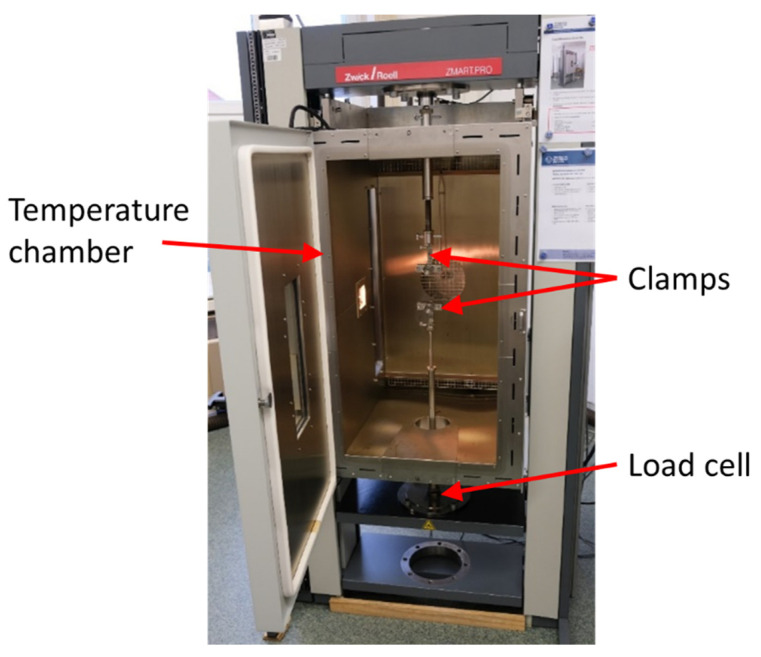
Zmart.Pro tensile testing machine with a connected temperature chamber.

Thermomechanical experiments were conducted on a Zwick & Roell Zmart.Pro tensile testing machine (ZwickRoell GmbH, Ulm, Germany) with a 500 N load cell and a connected temperature chamber with active nitrogen cooling, capable of performing mechanical testing under controlled temperature with a heat rate of 5 K/min (Figure 7). Specimens were clamped with a free length of 100 mm and a preload force of 0.1 N (1.4 MPa). To minimize temperature influences from the chamber toward the external load cell, a custom-built connecting rod with a reduced diameter was used to connect the lower clamp with the load cell.

Isothermal tensile tests were conducted at room temperature in order to capture martensite properties. Both as-delivered material with pre-strain as well as unstrained material were tested. The latter was obtained by heating pre-strained material up once to 130 °C under load-free condition. For measuring the austenite properties, unstrained SMA wires were clamped at 130 °C in order to avoid unwanted SMA contraction before the actual testing procedure. The tensile test was then conducted at 130 °C.

To estimate the maximum usable contraction of the SMA wires upon activation, UPFR tests [38] were carried out. The SMA wire was clamped within the temperature chamber at room temperature at a free length of *l*_0_ = 200 mm, with the load cell set to a low, constant holding force of 0.1 N. As the temperature chamber heated up, the wire began to contract, exerting a force on the clamps. The machine automatically changed the position of the mobile clamp in order to restore the initial constant holding force. The clamp position data were then used to measure the deformation of the wire. The SMA wire contraction Δl was then calculated using Equation (1). 

#### 2.2.2. Fiber-Rubber Composite Characterization

Tensile tests of single yarns were conducted based on ISO 3341 [39], enabling the determination of the elastic modulus of the 410 tex rovings. The properties of the 2 × 136 tex twisted twin-yarn were obtained from a technical data sheet provided by CULIMETA^®^. 

For estimating the mechanical properties of the silicone, tensile tests according to DIN EN ISO 527-4 [40] were performed. The specimens were produced by molding a rubber plate and cutting out the desired specimen geometry with ROFIN DC035 CO_2_-Laser. Before molding, the silicone requires thorough degassing in order to avoid defects caused by air bubbles. Since the stress–strain curve of rubbers is usually nonlinear, an elastic modulus cannot be determined accurately. The elastic modulus obtained from the beginning of the tensile test (for strains ε ≤ 100%) offers a rough estimation. Therefore, the stress–strain data from the tensile tests are used for calibrating the rubber material model. 

Since the primary deformation mode of the IFRC used in this work is bending, the flexural modulus is the most relevant property for validating the mechanical behavior of the simulation model with regard to the manufactured specimens. Therefore, the bending behavior of the FRC was investigated by using a four-point-bending test on a Zwick & Roell^®^ Z100 testing machine (ZwickRoell GmbH, Ulm, Germany) based on DIN EN ISO 14125 [41].

#### 2.2.3. Activation Test Setup

**Figure 8:**
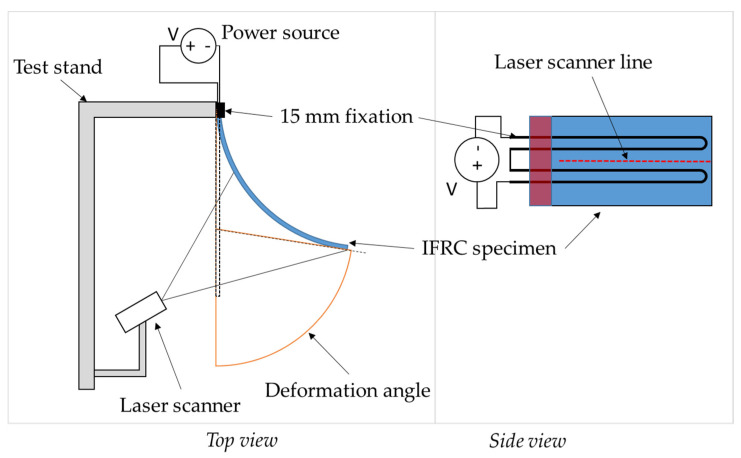
Deformation measurement test stand in top and side views.

The test setup for deformation measurements of the IFRC specimen is set such that the specimen deforms parallel to the ground, minimizing the influence of gravity (Figure 8). The IFRC specimen is clamped at one end only. The SMA wires are fixed from the external side by clamping between screws and ring washers. The deformation is measured with a laser scanner (LJ-V7200, Keyence GmbH, Osaka, Japan) pointed at the backside of the specimen. The scanner has a range of 152 to 248 mm and a resolution of up to 1.0 µm. The scanner’s CCD line sensor captures the bent shape of the IFRC sample as depicted in Figure 8, from which the deformation angle is derived in MATLAB (R2021b, The MathWorks Inc., Natick (MA), USA). The deformation angle is used as a measure of the achievable deformation and is obtained by applying a tangent to the free end of the arc-shaped deformed sample. A laboratory power supply unit Rohde&Schwarz HMP4040 is used as energy input, supplying the SMA wires with electric current through alligator clamps. The voltage during heating is set to 14 V, resulting in a current of ~2 A for the looped SMA wire with a total length of about 240 mm. A cycle time of 10 s is set for the activation phase and 10 s for deactivation, with a total of 10 cycles.

### 2.3. Simulation Model Development

#### 2.3.1. Geometry Modeling 

**Figure 9:**
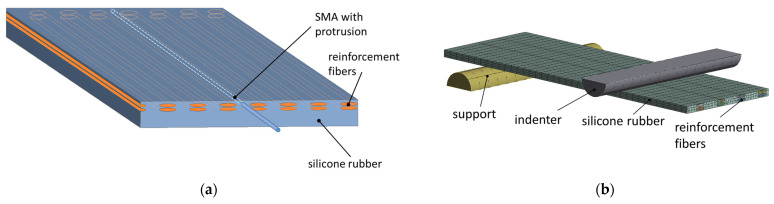
(**a**) The IFRC simulation model concept and (**b**) the four-point-bending model of the FRC without SMA and with symmetry.

The FRC plate is modeled in ANSYS^®^ as a silicone block with a cylindrical channel for the SMA and elliptical cavities for the reinforcement fibers. The SMA wire is modeled as a cylinder positioned within the channel without structural connection to the silicone. The fibers are modeled as cylinders with an elliptical cross section and placed inside the matching cavities. The fiber dimension parameters were obtained from a microscopy image of the real cross section and are listed in Table 2. The Var1 and Var2 specimens are modeled accordingly. For Var1, the cross-sectional areas of the two parts of the twisted twin-yarn are combined into one, neglecting the twist in the longitudinal direction. The coinciding nodes of the fiber geometry and the silicone are merged, assuming ideal interface bonding. All components are meshed with 3D elements. The model concept is displayed in Figure 9a. For modeling the four-point-bending behavior, the SMA wire was neglected and only fibers and rubber matrix were modeled, while using the symmetry of the setup by modeling only half of the structure (Figure 9b).

The plate length was reduced by 15 mm from the real length, replicating the fixation area of the activation test setup (cf. Figure 8). Since the SMA wire is still able to contract within this fixed 15 mm part of the specimen and thus has a significant influence on the overall SMA contraction, the wire was modeled 15 mm longer, protruding from the fixed side of the plate, matching the length of the wire in the real specimen. To increase the stability and efficiency of the model, several simplifications were made:

- As preliminary simulations have shown, the influence of the warp yarns on the bending behavior in the weft direction is small, while the modeling and computational effort for adding the warp yarns is high. Therefore, only the weft yarns are modeled, which reduces the computation time by a factor of ~100.
- A 20.75 mm wide section of the total structure with only one SMA wire is modeled. It is assumed that the actuation force of the SMA spreads evenly over the width of the specimen. The number of reinforcement fibers is reduced accordingly, reducing the total amount of elements and therefore the computing effort.
- The loop-shaped fixation of the SMA wire on the free end is neglected. Due to the necessity to pre-strain the wires during the simulation (see Section 2.3.2 and Section 2.3.3), a bent configuration is not feasible with the current SMA material model. Instead, the free end of the SMA is fixed to a small block connected to the free end of the FRC structure (see Section 2.3.3).

The mesh fineness was determined by performing a convergence analysis with varying numbers elements. The chosen number of elements is a compromise of accuracy and computational time and can be found in Table 3.

#### 2.3.2. Material Modeling

The SMA wire is assigned a material model based on the work of Auricchio and Souza [42,43], which is able to describe the OWSME. The 3D thermomechanical model for stress-induced solid phase transformations is based on the free energy potential

(4) $$\Psi \left(\varepsilon ,T,\varepsilon_{tr}\right)=\frac{1}{2}\left(\varepsilon -\varepsilon_{tr}\right):D:\left(\varepsilon -\varepsilon_{tr}\right)+\tau_{M}\left(T\right)\|{\varepsilon^{\prime }}_{tr}\|+\frac{1}{2}h\|{\varepsilon^{\prime }}_{tr}\|{}^{2}+I_{{\varepsilon^{\prime }}_{tr}}\left({\varepsilon^{\prime }}_{tr}\right)$$

where ***D*** is the material elastic stiffness tensor; *ε* is the total strain; $\varepsilon_{tr}$ is the total transformation strain; ${\varepsilon \prime }_{tr}$ is the deviatoric transformation strain; $\tau_{M}$(*T*) is a function of the temperature with $\beta \left(T-T_{0}\right)$; β is a material parameter; *T* is the temperature; *T*_0_ is the temperature below which no austenite is present in a stress-free state; *h* is a material parameter related to material hardening during the transformation; and $I_{{\varepsilon^{\prime }}_{tr}}$ is an indicator function describing the transformation, which is 0 for 0 ≤ $\left\|{\varepsilon^{\prime }}_{tr}\right\|$ ≤ $\varepsilon_{L}$ and becomes +∞ otherwise, with $\varepsilon_{L}$ as the maximum transformation strain. The stresses are then calculated with

(5) $$\sigma =\frac{\partial \Psi }{\partial \varepsilon }$$

(6) $$X_{tr}=-\frac{\partial \Psi }{\partial \varepsilon \prime_{tr}}$$

where $X_{tr}$ is the transformation stress. The Lode factor *m* describes the asymmetry between tension and compression behavior. The model reproduces the primary features of shape memory materials in a 3D stress state and is defined by nine parameters (Table 4). 

The material parameters are obtained from the thermomechanical characterization described in Section 2.2.1 by iterative fitting of the parameters to the measurement curves. To model the shape memory effect with this material model, the pre-strain must be applied within the simulation, prior to temperature activation, as is described in Section 2.3.3.

The rubber block was assigned an isotropic hyperelastic material model by Yeoh et al. [44], implemented within ANSYS^®^. The model uses a reduced polymer form of the strain energy potential with

(7) $$W=\overset{N}{\underset{i=1}{\sum }}C_{i0}{\left(\bar{I_{1}}-3\right)}^{i}+\overset{N}{\underset{k=1}{\sum }}\frac{1}{d_{k}}{\left(J-1\right)}^{2k}$$

where *W* is the strain energy potential; $\bar{I_{1}}$ is the first deviatoric strain invariant; *J* is the determinant of the elastic deformation gradient; and *N*, $C_{i0}$, and $d_{k}$ are material constants. The model is used with *n* = 3, requiring the constants $C_{10}$, $C_{20}$, $C_{30}$, $d_{1}$, $d_{2}$, and $d_{3}$. The constants are obtained by curve fitting of the model with the data obtained from the silicone tensile tests. 

The reinforcement fibers are assigned an orthotropic elastic material model, where the stiffness values of the respective fiber directions are inserted into the elastic coefficient matrix, with the elastic modulus in the main fiber direction *x* taken from the tensile tests of the GF material. The second and third directions, *y* and *z*, are defined for numerical stability. All material model parameters used in this work are displayed in Section 3.4.

#### 2.3.3. Boundary Conditions 

**Figure 10:**
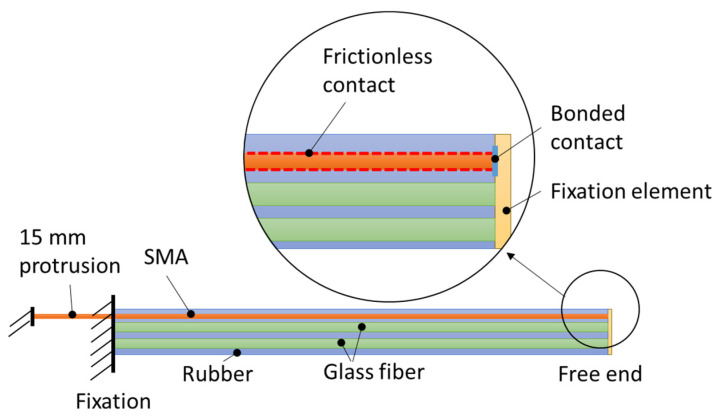
IFRC model boundary condition.

The boundary conditions used in the model are displayed in Figure 10. The rubber structure, the SMA wire, and the reinforcement fibers are fixed at one end, with the other end remaining free. The interaction of the SMA wire with its surrounding silicone channel is modeled with a frictionless contact formulation. Next, the connection of the SMA wire to the free end of the beam is modeled with a bonded contact formulation, tying the degrees of freedom of the SMA wires’ end nodes to the small external block connected to the free end of the beam. As preliminary investigations revealed, connecting the SMA wire directly to the rubber results in instabilities caused by large local deformations. 

The SMA wire is pre-strained in a preliminary load step by assigning a pre-strain force of 20 N to the free end. In a second preliminary load step, the pre-strain force is discarded, allowing the SMA wire to deform back elastically, leaving the wire in a state of detwinned martensite. This is followed by activating the respective bonded contact formulation such that the free end is connected to the FRC structure. Afterward, a temperature load step is assigned to the SMA wire, where the temperature is ramped up from 70 to 130 °C. This initiates the contraction of the wire, which in turn results in the bending of the IFRC structure. Analogous to the activation tests, the deformation angle of the free end is used for evaluating the resulting deformation.

## 3. Results

In this section, the results of the experiments, characterization tests, and simulations are presented. First, the results from the SMA material characterization are presented and some distinctive features are specified. Next, the results and obtained material parameters from the fiber, rubber, and fiber-rubber characterization experiments are listed. Then, the results obtained from the IFRC activation tests are presented and evaluated. Finally, the simulation model is calibrated with the data from the characterization experiments and validated by a comparison of its results with the activation test data.

### 3.1. SMA Characterization

Subsequently, the results from the characterization tests regarding DSC, isothermal tensile tests, and UPFR tests are presented, as well as the summarized SMA material properties obtained from the tests.

#### 3.1.1. DSC

**Figure 11:**
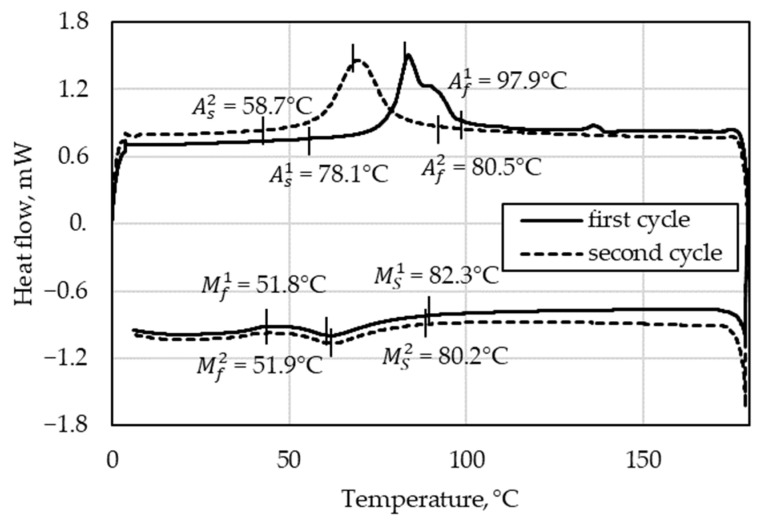
DSC test results of the first (superscript 1) and second (superscript 2) heating cycles of the SMA material.

The resulting heat flow curves are displayed in Figure 11. The positive peaks indicate the endothermic M→A transformation, where additional heat is required for heating the specimen, thus resulting in a positive latent heat, and vice versa for the exothermic A→M transformation. The pre-strain is removed during the heating phase of the first cycle, which explains the disparity between the first and second cycles during heating and confirms both the existence of the OWSME within the material and the necessity to pre-strain the material before usage as a wire actuator. The subsequent tests further affirm this assessment. The transformation temperatures obtained from the DSC serve as an estimation of the activation temperature range.

#### 3.1.2. Isothermal Tensile Tests

**Figure 12:**
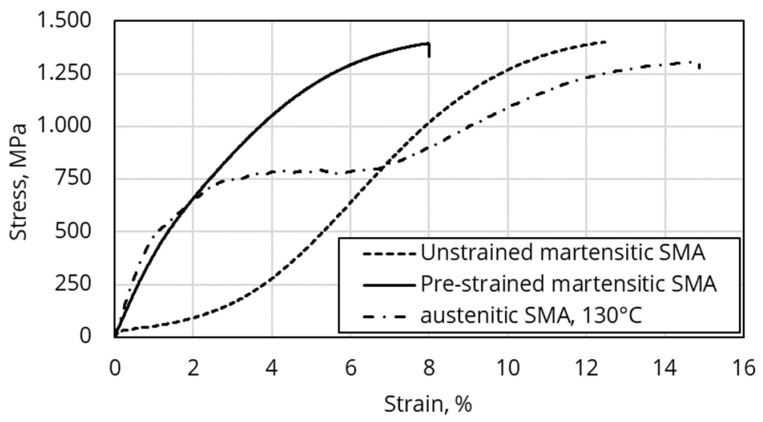
Tensile test of pre-strained as well as unstrained martensitic SMA at room temperature and austenitic SMA at 130 °C.

Figure 12 shows two exemplary stress–strain curves of both a pre-strained and an unstrained martensitic SMA specimen, as well as the curve of one austenitic specimen. From the pre-strain-free martensite and the austenite, the elastic moduli *E_M_* and *E_A_* are obtained by applying a tangent through the point of origin. For usage in the SMA material model, it is important to use the pre-strain-free data for *E_M_* in order to capture the material behavior in its initial, stress-free state. Furthermore, from the curve of the pre-strain-free specimen, the range and required stress of martensitic detwinning can be identified. For a pre-strain of 4%, a pre-stress of about 250 MPa (~18 N for d = 0.3 mm) is required. This stress value is used for the pre-strain loading of the simulation model in Section 3.4.

#### 3.1.3. Uniaxial Pre-Strain and Free Recovery (UPFR) Test

**Figure 13:**
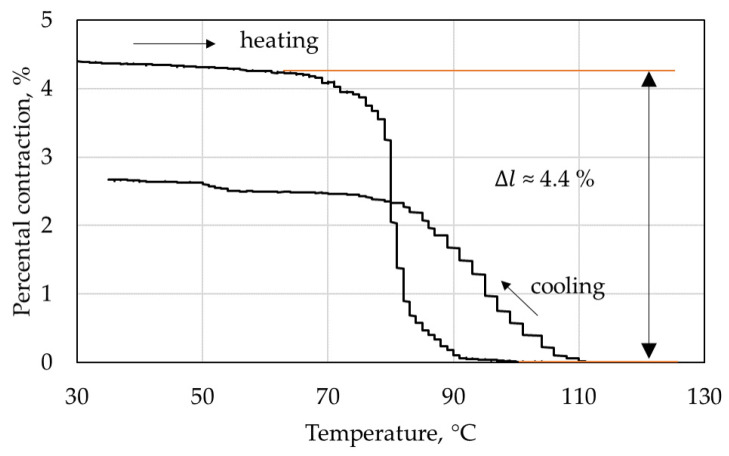
UPFR test of the SMA wire material.

An exemplary curve of an SMA wire specimen during the UPFR test is displayed in Figure 13, with the arrow indicating the directions of the heating and cooling processes. Upon reaching 70 °C, the SMA wire contracts by a significant amount and finishes its contraction around 90 °C. The maximum SMA wire contraction Δ*l* obtained from the data is found to be 4.4 ± 0.2%. It is worth noting that the effective contraction of a structurally integrated SMA wire is impeded by the structural stiffness of the surrounding material, as was described in Section 1.1.3. Therefore, the obtained value for Δ*l* is regarded as a maximum value. Furthermore, a partial setback upon deactivation can be observed. During cooling, the SMA wire extends again by a significant amount. As was confirmed by the manufacturer, the heat treatment during manufacturing resulted in a partial two-way effect. This effect has a significant influence on the setback behavior of the IFRC structures, enabling larger achievable setback angles (cf. Section 3.4).

#### 3.1.4. SMA Material Properties

The obtained thermomechanical properties of the SMA wire material are listed in Table 5. As mentioned before, the martensitic modulus was obtained from unstrained (i.e., twinned) specimens.

### 3.2. Fiber, Rubber, and Fiber-Rubber Composite Characterization

The material properties of the silicone rubber and GF yarn materials are displayed in Table 6 and Table 7, respectively. As expected, the stiff GF rovings dominate the tensile properties of the FRC, which indicates highly anisotropic behavior for the FRC material. The results of the four-point-bending test in comparison to the simulation results are displayed in Section 3.4.

### 3.3. Activation Test

**Figure 14:**
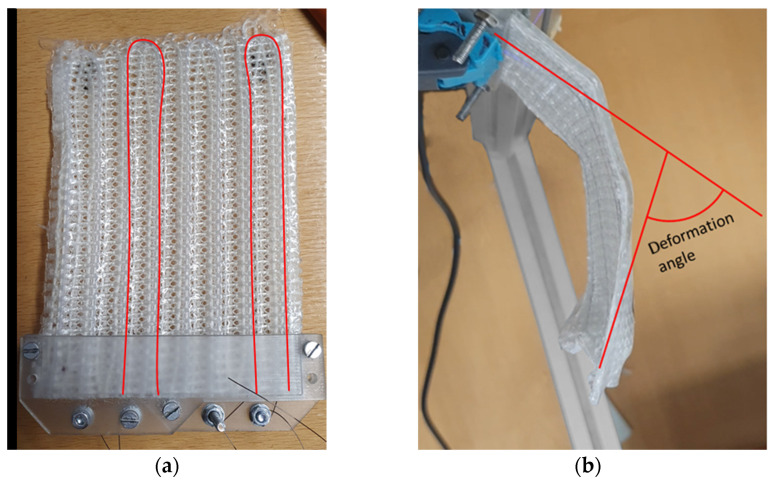
(**a**) IFRC specimen Var1 with two integrated SMA loops and (**b**) deformed IFRC specimen Var1 with two activated SMA loops and a geometric approximation of the deformation angle.

Both Var1 and Var2 were equipped with two SMA loops, resulting in four parallel SMA wire strands (Figure 14a). The specimen showed significant deformation behavior (Figure 14b), with a maximum deformation angle of 52.8° for Var1 and 23.8° for Var 2. Several effects can be observed during the activation:

1. The free ends of the plate curl in the area of the SMA redirection. This effect decreases with a larger number of SMA wires within the structure. With more SMA wires, the force required for deforming the whole structure is distributed more evenly over the whole structure. Therefore, the force transmitted onto the structure in the redirection area of each individual wire becomes lower.
2. As expected, increasing or decreasing the number of parallel SMA wires within the sample increases or decreases the achievable deformations, respectively. However, decreasing the number of parallel SMA loops to a value below a certain value (below two for Var1) leads to a local curling of the free end of the plate, with barely any global bending deformation. The actuation force of the SMA is concentrated on the small area at the free end, where the SMA is fixed by its loop shape. The stiffness in the weft direction is not high enough to distribute the load evenly over the whole plate, curling the area locally instead.
3. The bent structure shows creases along its length, diverting from the ideal arc shape. The creases are assumed to be caused by local defects of the interface between fiber and matrix.

**Figure 15:**
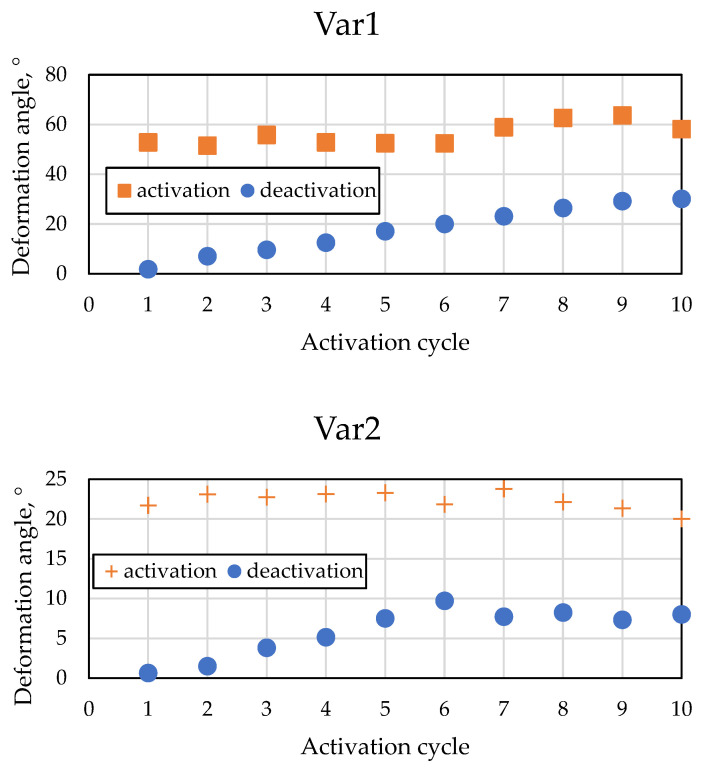
Activation test results of the IFRC specimens Var1 and Var2.

The specimen with one loop showed the aforementioned local curling of the free end, which resulted in deformations close to no bending. Furthermore, a specimen with six loops showed a large deformation, up to 180°, which could not be captured with the laser triangulation setup. The resulting deformation angles after activation and deactivation are displayed in Figure 15.

Due to the overall irregular deformation shape of the specimens, their uneven surface, and the local curling at the free ends, evaluating the deformation angle from the measurement results was challenging. Therefore, the estimated deformation angles can only serve as a rough estimation of the real deformation behavior that was achieved. However, several effects can be observed:

1. For both specimens, the residual angle after deactivation increases with each cycle. This effect is typical for SMA-driven composites and was observed in previous works, e.g., by Ashir et al. [30,45]. Additionally, it can be assumed that defects caused by the delamination of fiber and rubber add to this effect.
2. The specimen Var1 shows significantly larger deformation angles. This confirms the assumption that structures with lower stiffness allow for larger deformations induced by the SMA.

### 3.4. Simulation Model Calibration and Validation

The material model parameters of the hyperelastic material model for the silicone, the SMA material model, and the anisotropic material model for fiber reinforcement are displayed in Table 8, Table 9 and Table 10, respectively. The parameters for the Yeoh hyperelastic material model were obtained by an automated curve fitting function implemented in ANSYS^®^, where the software iteratively determines the parameters for a good agreement with the provided experimental data. Since no compression data were entered, the bulk modulus parameters *d*_1_*–d*_3_ are determined as zero. No details are given in the ANSYS^®^ instructional appendix on how the model processes these zero values, which would lead to division by zero in Equation (7), but it can be assumed that the bulk terms of Equation (7) are neglected.

The same material model was used for both GF materials. The stiffness parameters of the GF material were calibrated iteratively by comparing the results of the four-point-bending test with the simulation. The starting value obtained from the tensile test data of the fiber material resulted in higher bending stiffness than in the experiment by a factor of ~10. It is thus assumed that for thin FRC specimens and due to the low rubber matrix stiffness, an effect similar to that in pure textiles occurs: the high tensile stiffness is no longer coupled to the bending stiffness in the specimen, resulting in an overestimation of the bending stiffness.

**Figure 16:**
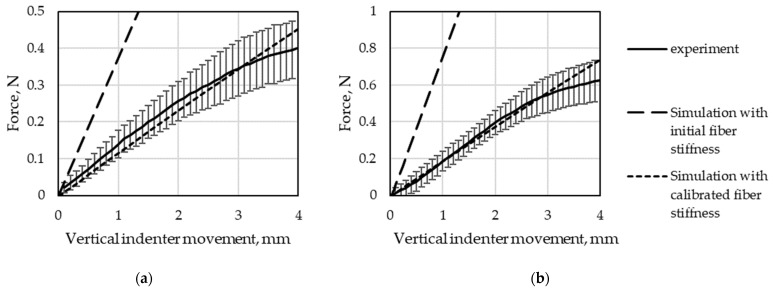
Four-point-bending test results from the simulation and experiment of FRC specimens for (**a**) Var1 and (**b**) Var2.

The results of the four-point-bending simulation conducted with the calibrated parameters for the fibers listed above are compared with the experimental results, as displayed in Figure 16.

**Figure 17:**
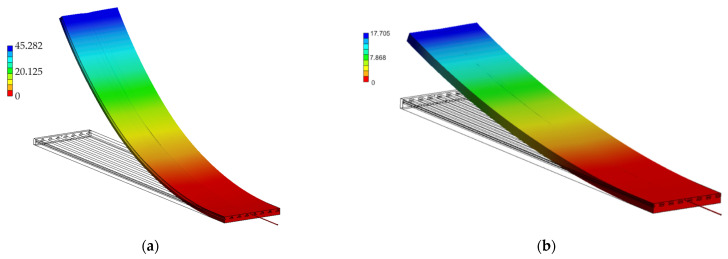
Simulation results of (**a**) Var1 and (**b**) Var2. The color coding denotes the vertical deformation.

**Figure 18:**
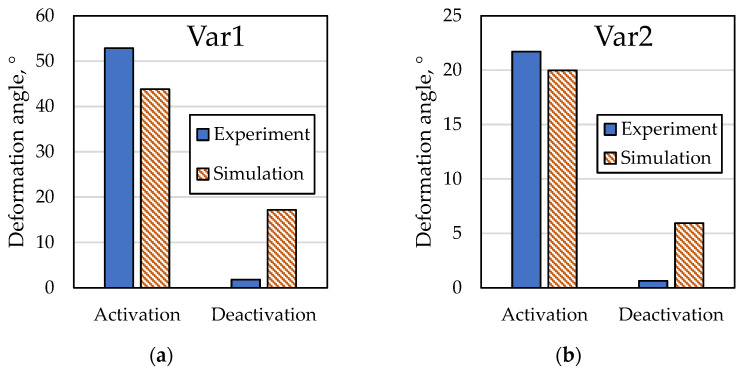
Comparison of deformation angles of simulation and experiment of (**a**) Var1 and (**b**) Var2.

The IFRC simulation model shows the expected arc-shaped bending deformation, as displayed in Figure 17, with a maximum deformation angle of 43.8° for Var1 and 20.0° for Var2. The achieved deformation angles of both simulation and experiment are displayed in Figure 18. The disparity of the results of Var1 is bigger than that of Var2. An explanation for this effect is the chosen activation temperature of 130 °C. As discussed earlier, the SMA cannot fully transform, due to the stiffness of the FRC blocking the full transformation. Heating the SMA further causes the transformation to continue, which seemingly causes numerical stability problems. However, this effect only appears when the SMA is combined with a structure that achieves high deformations during activation, as is the case with Var1. For Var2, with higher stiffness and lower deformations, finding a stable solution appears easier, leading to simulation results that are closer to the experimental ones. Therefore, a compromise between a stable solution and an as-high-as-possible activation temperature is chosen for Var1, leading to a discrepancy of 9.0° for the activation. 

The deactivation experiments and simulations show a disparity, which is assumed to be related to the partial two-way shape memory effect (TWSME, cf. Section 1.1.1) inherent with the SMA material. This effect cannot be depicted accurately with the SMA material model. Upon cooling, the SMA deforms back by a considerable amount without the FRC having to apply an additional force, thus supporting the overall backward movement. Here, the advanced SMA model by Woodworth et al. [45] is a promising approach to replicating this behavior.

## 4. Conclusions

The simulation model is capable of capturing the bending behavior of SMA-driven IFRC structures with overall good agreement with the measurements conducted in this work. The model enables the user to estimate the influence of many different parameters on the deformation behavior, such as the used materials; the amount and positioning of the reinforcement fibers; the overall geometry; and additional external factors, such as the weight of the structure or contact interactions with solid bodies. Thus, the model can be used for the development and dimensioning of future IFRC structures and applications.

Several aspects make model development a challenging task. A significant matter is the fixation of the SMA at the free end of the beam. As the activation tests have shown, the stiffness of the IFRC structure at the free end of the plate is too small for transmitting the SMA force onto the whole structure when using only one SMA loop, resulting in curling of the plate edges. To apply the necessary pre-straining into the SMA wire, the model was simplified with a straight SMA wire. With a more advanced model, this drawback could be replaced by the SMA material model. Such an approach was developed recently [46].

For the bending structures investigated in this work, the 90° fibers had a low influence on the deformation behavior. However, for future investigations of, e.g., more complex structures and deformation patterns, the necessity of modeling the 90° fibers should again be verified.

An undesirable effect is the local creasing of the bent structure, which leads to uneven bending arcs. The creasing is assumed to be caused by defects of the interface between rubber matrix and glass fiber. The high viscosity of the silicone impedes the full impregnation of the fiber bundles during the VARI process. Therefore, this aspect should be improved in the future.

Future investigations will use the established model for investigating the influences of both geometrical and material parameters on the deformation behavior, as well as the development of more complex geometries and mechanisms. For this, further developments of the model, such as using advanced SMA material models or the application of macroscopic formulations for the IFRC behavior, will help to increase the model’s accuracy and capabilities.

## Figures and Tables

**Table 1:** Composite layup for the production of fiber-rubber composites.

Layer, Top to Bottom	Description
1 Vacuum foil	Seals the layup air-tight
2 Flow media	Supports the distribution of silicone and vacuum
3 Peel ply	Detaches the release film from the specimen
4 Reinforcement textile	Glass fiber weave cuttings
5 Mold	Sheet metal supporting the composite forming

**Table 2:** Geometry modeling parameters.

Geometry Parameter	Value
Plate length × width × height (mm³)	105 × 20.75 × 2.0 (Var1)/2.5 (Var2)
410 tex fiber ellipsis radius a (mm)	0.8
410 tex fiber ellipsis radius b (mm)	0.17
2 × 136 tex fiber ellipsis radius a (mm)	0.37
2 × 136 tex fiber ellipsis radius b (mm)	0.18
Number of fibers	7.25

**Table 3:** Mesh details.

Component	Element Type	Element Number
410 tex fiber	Hexaeder	400
2 × 136 tex fiber	Hexaeder	120
Rubber matrix	Hexaeder	14,520
SMA wire	Hexaeder	60
Indenter and support	Hexaeder	61
Total		17,168

**Table 4:** SMA material model parameters.

Parameter	Meaning	Property and Unit
MP	*E_A_*	Austenite modulus, MPa
EX	ϑ	Poisson ratio
C1	*h*	Hardening parameter, MPa
C2	*T*_0_	reference temperature, °C
C3	*R*	Elastic stress limit, MPa
C4	*β*	Temperature scaling, MPa/K
C5	$\varepsilon_{L}$	Maximum transformation strain, %
C6	*E_M_*	Martensite modulus, MPa
C7	*m*	Lode factor

**Table 5:** Material properties of the NiTi SMA wire material.

Property	Value		Value	
Alloying components * (wt%)	Ni	54.8	Nb	0.0001
	Cu	0.0005	Co	0.0002
	Fe	0.013	Ti	Bal
Wire diameter (mm)	d = 0.305			
Phase transition temperatures (°C)	A_s_ = 78.1		M_s_ = 82.3	
	A_f_ = 97.9		M_f_ = 51.8	
Maximum actuation contraction (%)	4.4 ± 0.2		-	
Young’s modulus (GPa)	E_M_ = 32.5		-	
	E_A_ = 53.6			
Density (kg/m³)	5000		-	

* Datasheet provided by SAES^®^ Getter.

**Table 6:** Properties of the silicone rubber material.

Property	Value
Elastic modulus (0–10% strain) (MPa)	1.1
Fracture stress (MPa)	4.5
Fracture strain (%)	86.8
Density * (g/cm³)	1.05

* Datasheet provided by ^®^ DOW CORNING.

**Table 7:** Properties of the glass fiber yarns used for the knitted fabrics.

410 Tex Glass Fiber Yarn	
Elastic modulus (GPa)	80.2 GPa
Yarn diameter (mm)	1.4
Yarn count/fineness (tex)	410
Density (g/cm³)	2.6 g/cm³
2 × 136 Tex Glass Fiber Twisted Twin-Yarn *	
Elastic modulus (GPa)	80.2 **
Yarn diameter (mm)	0.4
Linear density (tex)	2 × 136 tex
Density (g/cm³)	2.6 g/cm³

* Datasheet provided by ^®^ CULIMETA; ** estimated value.

**Table 8:** Material parameter set for the hyperelastic material of the silicone.

Material Model Parameter	Value
$C_{10}$ (Pa)	770,623.97
$C_{20}$ (Pa)	−386,308.55
$C_{30}$ (Pa)	196,852.40
$d_{1}$ (Pa^−1^)	0
$d_{2}$ (Pa^−1^)	0
$d_{3}$ (Pa^−1^)	0

**Table 9:** Material parameter set for the SMA material.

Parameter	Unit	Value	
		Room Temperature (Room Temperature)	High Temperature (130 °C)
MP	MPa	25,000	60,000
EX	-	0.3	0.3
C1	MPa	610	2534.4
C2	°C	70	70
C3	MPa	52	184.3
C4	MPa/K	7	4525
C5	%	0.0355	0.0572
C6	MPa	20,000	35,000
C7	-	0	0

**Table 10:** Material parameter set for the anisotropic fiber material.

Parameter	Value
$E_{x}$ (GPa)	5.0
$E_{y}$ (GPa)	0.04
$E_{z}$ (GPa)	0.04
$\vartheta_{xy}$	0.22
$\vartheta_{yz}$	0.22
$\vartheta_{xz}$	0.22
$G_{xy}$ (GPa)	0.04
$G_{yz}$ (GPa)	0.02
$G_{xz}$ (GPa)	0.04

## Data Availability

The data presented in this study are available on request from the corresponding author.
